# Global Inequities in Diabetes Technology and Insulin Access and Glycemic Outcomes

**DOI:** 10.1001/jamanetworkopen.2025.28933

**Published:** 2025-08-27

**Authors:** Alzbeta Santova, Martin de Bock, Stefanie Lanzinger, Ellen B. Goldbloom, Natasa Bratina, Consuelo Barcala, Doha Alhomaidah, Arunkumar R. Pande, Pravesh Kumar Guness, Iveta Dzivite-Krisane, Catarina Limbert, Zdenek Sumnik

**Affiliations:** 1 Department of Pediatrics, Motol University Hospital and Second Faculty of Medicine, Prague, Czechia; 2 First Faculty of Medicine, Charles University, Prague, Czechia; 3 Department of Pediatrics, University of Otago, Christchurch, New Zealand; 4 Institute of Epidemiology and Medical Biometry, Ulm University, Ulm, Germany; 5 German Center for Diabetes Research, Munich-Neuherberg, Germany; 6 Department of Pediatrics, Faculty of Medicine, University of Ottawa, Ottawa, Ontario, Canada; 7 Division of Endocrinology, Children’s Hospital of Eastern Ontario, Ottawa, Canada; 8 Children’s Hospital of Eastern Ontario Research Institute, Ottawa, Canada; 9 Department of Pediatric Endocrinology, Diabetes and Metabolic Diseases, University Children’s Hospital, University Medical Centre Ljubljana, Ljubljana, Slovenia; 10 Department of Nutrition and Diabetes, Hospital de Pediatría S.A.M.I.C. Prof. Dr. Juan P. Garrahan, Buenos Aires, Argentina; 11 Pediatric Endocrine and Diabetes Unit, Pediatric Department, Farwaniya Hospital, Al Farwaniyah Governorate, Kuwait; 12 Department of Endocrinology, Diabetes and Metabolism, Health City Vistaar Hospital, Lucknow, India; 13 Department of Endocrinology, Diabetes and Metabolism, Lucknow Endocrine Diabetes and Thyroid Clinic, Lucknow, India; 14 T1Diams, Quatre Bornes, Mauritius; 15 Medecine de Semaine Diabétologie, Groupe Hospitalier Est Reunion, Saint Benoit, France; 16 Children’s Endocrinology Unit, Children’s University Hospital and Riga Stradiņš University, Riga, Latvia; 17 Unit of Pediatric Endocrinology and Diabetes, Hospital Dona Estefania, Lisbon, Portugal; 18 Comprehensive Health Research Centre, NOVA Medical School, NOVA University of Lisbon, Lisbon, Portugal

## Abstract

**Importance:**

Advanced diabetes technologies such as continuous glucose monitoring (CGM), continuous subcutaneous insulin infusion (insulin pumps [CSII]), and glucometers alongside insulin access represent the criterion standard for managing type 1 diabetes (T1D) in children. Global disparities in their access and reimbursement may be associated with glycemic outcomes.

**Objective:**

To describe how accessibility and reimbursement of advanced diabetes technologies and insulin are associated with glycated hemoglobin (HbA_1c_) levels in centers participating in the SWEET initiative, an international pediatric diabetes registry.

**Design, Setting, and Participants:**

This global multicenter cross-sectional study collected data from 81 centers in 56 countries. Web-based questionnaires were distributed to representatives of all 121 pediatric diabetes centers participating in the SWEET initiative from March 1 to May 31, 2024, and used to map accessibility of and reimbursement for CGM, CSII, glucometers, and insulin. Reimbursement data were compared with HbA_1c_ levels using the SWEET Study dataset. Participants included 42 349 children with T1D.

**Exposures:**

Responses were categorized into 4 groups based on the extent of reimbursement for diabetes technologies and insulin.

**Main Outcomes and Measures:**

Mean HbA_1c_ levels across centers calculated from measurements current as of December 31, 2023, analyzed by categories of accessibility of and reimbursement for diabetes technologies and insulin.

**Results:**

Data collected from 81 of 121 SWEET centers (67%) across 56 countries included HbA_1c_ levels from 42 349 children with T1D (22 021 male [52%]; mean [SD] age, 14.3 [4.4] years; mean [SD] diabetes duration, 6.0 [4.2] years). Universal access with complete reimbursement for all technologies and insulin was reported by 32 centers from 19 countries, while 8 countries reported no reimbursement for any technologies or insulin. Centers with full reimbursement for CSII, CGM, glucometers, and insulin showed mean HbA_1c_ levels of 7.62% (95% CI, 7.59%-7.64%) to 7.75% (95% CI, 7.73%-7.77%) compared with 9.65% (95% CI, 9.55%-9.71%) to 10.49% (95% CI, 10.40%-10.58%) in centers with no reimbursement and/or no availability (*P* < .001 for all items).

**Conclusions and Relevance:**

This cross-sectional study found that HbA_1c_ levels were associated with the accessibility of modern diabetes technologies and insulin. Efforts to ensure universal accessibility are required to reduce global inequities and glycemic outcomes for children with T1D.

## Key Points

**Question:**

How is global disparity in access to diabetes technologies and insulin associated with glycemic outcomes in children with type 1 diabetes (T1D)?

**Findings:**

This cross-sectional study collated data regarding the accessibility and reimbursement of diabetes technologies and insulin from 81 centers across 56 countries, inclusive of 42 349 children with T1D. Significant global disparity and an association between glycemic outcomes and the accessibility of diabetes technologies and insulin were found.

**Meaning:**

Global efforts must be made to ensure universal accessibility to insulin and diabetes technologies and thereby improve disparity in glycemic outcomes for children with T1D.

## Teaser

This cross-sectional study estimates the association of accessibility and reimbursement for diabetes technologies and insulin with glycemic control among children with type 1 diabetes using data from the global SWEET Study dataset.

## Introduction

Advanced diabetes technologies are now considered the criterion standard for the management of type 1 diabetes (T1D), including in children.^1,2^ This recommendation is based on the numerous studies that demonstrate improved glycemia and quality of life.^3,4^ Rapid advancement in diabetes technologies,^5^ including the implementation of hybrid closed loop systems (HCL) integrating insulin dosing using a continuous subcutaneous insulin infusion (insulin pump [CSII]) and continuous glucose monitoring (CGM), has resulted in global disparities in terms of access^6,7^ and likely resultant glycemic inequity.^8,9^ Consequently, the Declaration of Lisbon recently articulated the ongoing commitment to enhance access to advanced diabetes technology and insulin.^10^

The SWEET initiative^11^ established in 2008 represents a global platform for benchmarking and networking between large pediatric diabetes centers.^12^ In 2023, 121 centers from 6 continents were sharing data on glycemic outcomes of children with T1D within the SWEET project. One of the missions of SWEET is to harmonize care to optimize outcomes of children with T1D worldwide.^13,14^ In line with this goal, 2 studies in 2009 and 2017 that mapped the accessibility of and reimbursement for insulin and diabetes technologies in Europe^6,15^ showed that the reimbursement strategy regarding modern technologies is very heterogeneous, even in developed countries. Despite some positive trends toward improved reimbursement observed between 2009 and 2017, access to these technologies remained limited in a significant proportion of countries because of the individual financial contribution requirement.^6^

The previous SWEET projects of 2009 and 2017 gathered data prior to the HCL era and did not address the association of accessibility to technologies with glycemic outcomes. Moreover, these studies mapped the reimbursement status exclusively in Europe. Therefore, we aimed to build on these data by describing the global accessibility and reimbursement of diabetes technologies and insulin for children with T1D in countries actively participating in the SWEET initiative and to compare these data with glycemic control as measured by glycated hemoglobin (HbA_1c_) levels using the data from the SWEET registry.

## Methods

### Study Design and Questionnaire Distribution

In this cross-sectional study, a web-based questionnaire was distributed by email to representatives of all SWEET centers via the SWEET coordination center in Hannover, Germany. Centers were asked to describe the accessibility of CGM, CSII with and without HCL functionality (including supplies), personal glucometers (including blood glucose reagent strips), and insulin. Categorized data for reimbursement of all items were then compared with HbA_1c_ levels using the SWEET dataset managed by the Institute of Epidemiology and Medical Biometry, Ulm University, Germany. The survey was conducted from March 1 to May 31, 2024, and mirrored the situation as of December 31, 2023. All centers participating in the SWEET project comply with current regulatory data protection security and ethics requirements, including data transfer agreement; ethics and institutional board approvals were not required for this questionnaire-based study mapping the reimbursement of diabetes technologies in participating countries. We followed the Strengthening the Reporting of Observational Studies in Epidemiology (STROBE) reporting guideline. Informed consent was not required for the use of deidentified registry data.

### Questionnaire

Given the potential differences in the accessibility and reimbursement of technologies and insulins across regions, states, territories, or provinces within a single country, the representatives of each center were asked to indicate whether they were responding for the entire country (preferred, if possible) or only for a specific region. The questionnaire consisted of both closed- and open-ended questions. The questions were focused on availability and the type of reimbursement and eligibility criteria for CGM, CSII with and without automation (including supplies), glucometers (including blood glucose reagent strips), and insulin. The participants also had the opportunity to describe any nonstandard reimbursement in narrative terms. The complete questionnaire is accessible in eAppendix 1 in Supplement 1.

### Categorization

Based on the answers from each center, countries (or regions, territories, or provinces) were categorized into 4 groups for each technology and insulin:

1. Full reimbursement: fully covered by the government and/or employers, for CGM allowing more than 90% of CGM use per year, for CSII and HCL allowing uninterrupted use, and for glucometers allowing glucose level measurement more than 5 times daily;
2. Limited reimbursement: copayments or geographical-, age-, and/or insurance-dependent differences within the country, province, or state or limiting indication criteria;
3. Out-of-pocket payment: no reimbursement, but possible to purchase the device or insulin; and
4. Supported by sponsors or no availability: no reimbursement, but unrestricted access to the technology and/or insulin is ensured through foundations or other similar sources, or no availability in the country.

The unavailability of HCL in a country, even with the full availability and reimbursement of CSII, automatically placed the country in a limited reimbursement category for CSII. When multiple centers from the same country provided responses on behalf of the entire country, the agreement in their responses was independently checked by the first (A.S.) and the senior (Z.S.) authors. For countries with more than 1 participating center, responses were consistent in all but 2 countries. These participants were subsequently contacted for further clarification. For confirmatory dataset, one of the SWEET corporate members (Medtronic Europe) was approached with a similarly structured questionnaire. Only data for European countries were available for validation.

### Glycemic Outcomes

HbA_1c_ levels were standardized to the reference range of the Diabetes Control and Complications Trial (4.0%-6.0% [20-42 mmol/mol]).^16^ Categorized data for all items were then compared with HbA_1c_ levels using the SWEET dataset. Glycemic outcomes were assessed by HbA_1c_ levels as the only objective glycemic outcome widely available in the SWEET database. To associate data on the accessibility and reimbursement of diabetes technologies and insulin with glycemic parameters, data of all children with T1D younger than 21 years in 2023 who were followed up in the centers that completed the survey were used. For each center, the means of participants’ HbA_1c_ levels during the most recent treatment year between 2019 and 2023 were calculated from all available values. Analysis using 2023 data only did not affect the results of the study (eTable 1 in Supplement 1). All contributing centers complied with current regulatory data protection security and ethics requirements.

### Statistical Analysis

Multivariable linear regression models were used to study HbA_1c_ levels in association with reimbursement categories. Models were adjusted for sex, age group (<6, 6 to <10, 10 to <14, 14 to <18, and ≥18 years), and diabetes duration (<2, 2 to <5, 5 to <10, and ≥10 years). Statistical analyses were conducted using SAS, version 9.4 (SAS Institute Inc), and 2-sided *P* < .05 was considered statistically significant. Results are presented as adjusted least squares means together with 95% CIs and were estimated using observed marginal distributions of covariates. Moreover, bubble plots were created to present HbA_1c_ levels in association with reimbursement separated by 6 continents (Africa, Asia, Australasia, Europe, Latin America, and North America).

## Results

The questionnaire was distributed to 121 SWEET centers from 64 countries. There were 81 responses (67% response rate) from 56 countries representing 88% of SWEET countries. HbA_1c_ data from 42 349 children with T1D (20 328 female [48%] and 22 021 male [52%]; mean [SD] age, 14.3 [4.4] years; mean [SD] diabetes duration, 6.0 [4.2] years) from these centers were available for analysis. The overall characteristics of the study group are described in eTable 2 in Supplement 1. SWEET Study Group members are listed in eAppendix 2 in Supplement 1.

Most responses were reported from Europe (39 centers from 26 countries), followed by Asia (19 centers from 12 countries), Latin and North America (14 centers from 10 countries), Africa (6 centers from 6 countries), and Australasia (3 centers from 2 countries). The list of the participating countries is included in the eTable 3 in Supplement 1). The responses from 48 of 56 countries (86%) covered the entire country, and the remaining 8 countries (Argentina, Canada, Ghana, Hong Kong, Italy, Morocco, the United Kingdom, and US,) reported for a part of the country only. The validation showed a minor discrepancy in CGM reimbursement in Bulgaria. The country study coordinator was therefore contacted and the status was clarified.

**Figure 1.**
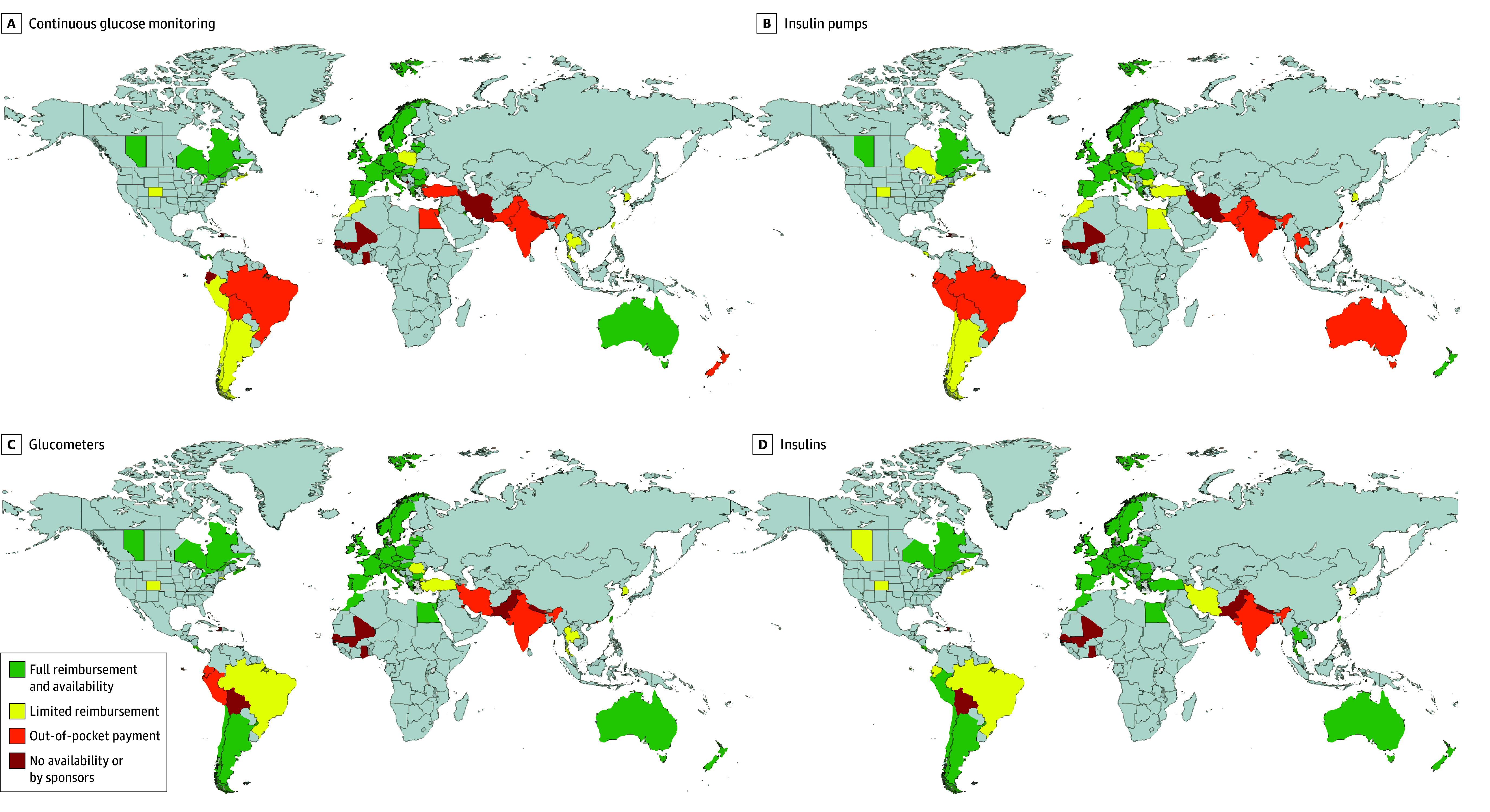
The Accessibility of and Reimbursement Type for Diabetes Technology in Different Countries Created with MapChart, version 6.7.1, April 29, 2025.

Full access to and reimbursement for all surveyed technologies and insulin were reported by 32 of 81 centers (40%) from 19 of 56 countries (34%). Conversely, none of the surveyed technologies nor insulin was reimbursed in 8 countries (Bolivia, Ghana, Haiti, India, Mali, Nepal, Pakistan, and Senegal). The availability of and type of reimbursement for technologies and insulin in individual countries are detailed in Figure 1 and eTable 2 in Supplement 1.

### CGM Availability and Reimbursement

Accessibility and reimbursement were most homogeneous in Europe, where full coverage was reported in 24 of 26 countries (92%). The limited reimbursement was noted in Poland, where significant copayment as high as 20% to 30% was required for all types of CGM. No access to this technology was reported in Montenegro.

High heterogeneity was seen in Asia and Latin and North America, where limited reimbursement was the most common. Substantial copayments as well as geographical differences were the most frequently reported limitations in Asia. In North America, copayments depending on the type of health insurance constituted the most common limitation reported. Full reimbursement was reported in Australia. An out-of-pocket payment was needed in New Zealand. Of the 6 participating countries in Africa, no availability of CGM was reported by four.

### Insulin Pump Availability and Reimbursement

In Europe, 19 of 26 countries (73%) reported full coverage of CSII including HCL. In the remaining 7 countries (Bulgaria, Croatia, Latvia, Lithuania, Montenegro, Poland, and Switzerland), some limitations in reimbursement were reported. The described limitations included copayments for CSII (Switzerland), copayments for insulin supplies (Poland), or copayments with other limitations such as age (Croatia and Lithuania). In Bulgaria, Latvia, and Montenegro, full coverage of CSII was reported but without coverage for HCL.

A heterogeneous situation was described in North America, where full reimbursement was noted in some Canadian provinces and limited reimbursement in the US and other Canadian provinces. In Latin America, limited reimbursement and out-of-pocket payments were mostly reported. The most common limitations included different copayments based on insurance types and strict indication criteria for initiation of the therapy.

A requirement for an out-of-pocket payment was the predominant response in Asian countries. None of the African countries reported full reimbursement of CSII. In contrast to CGM accessibility, an out-of-pocket payment requirement was recorded in Australia for CSII. Full reimbursement was reported in New Zealand.

### Glucometer Availability and Reimbursement

Full reimbursement for glucometers, including glucometer strips, was stated in most countries in Europe and Australia. In Europe, only Romania reported limited reimbursement in terms of a limited quantity of reimbursed strips (300 strips per 3 months for children with T1D without CGM, 100 strips per 3 months for children with T1D using CGM). In Asia and Latin and North America, reported reimbursement was variable. Full reimbursement was reported by 3 African countries (Egypt, Mauritius, and Morocco), while no reimbursement and dependence on sponsors were described in Ghana, Mali, and Senegal.

### Insulin Availability and Reimbursement

Insulin was reported as fully reimbursed in all participating countries in Europe, Australia, and New Zealand. Full reimbursement was reported in several Asian countries (Hong Kong, Israel, Kuwait, Maldives, Taiwan, Thailand, and Turkiye), and limited reimbursement was reported in Iran and South Korea, out-of-pocket purchases in India, and dependence on sponsors in Nepal and Pakistan.

Similarly, in North and Latin America, full reimbursement was the most frequent; however, there were some limitations in reimbursement described in several countries (Ecuador, Brazil, and the US and some Canadian provinces), particularly concerning copayments with various types of health insurance. Availability through sponsorship was reported in Haiti and Bolivia. Insulin was fully reimbursed in some parts of Africa (Egypt, Mauritius, and Morocco); however, access to insulin through sponsorship was reported by Ghana, Mali, and Senegal.

### HbA_1c_ Levels by Type of Reimbursement

**Figure 2.**
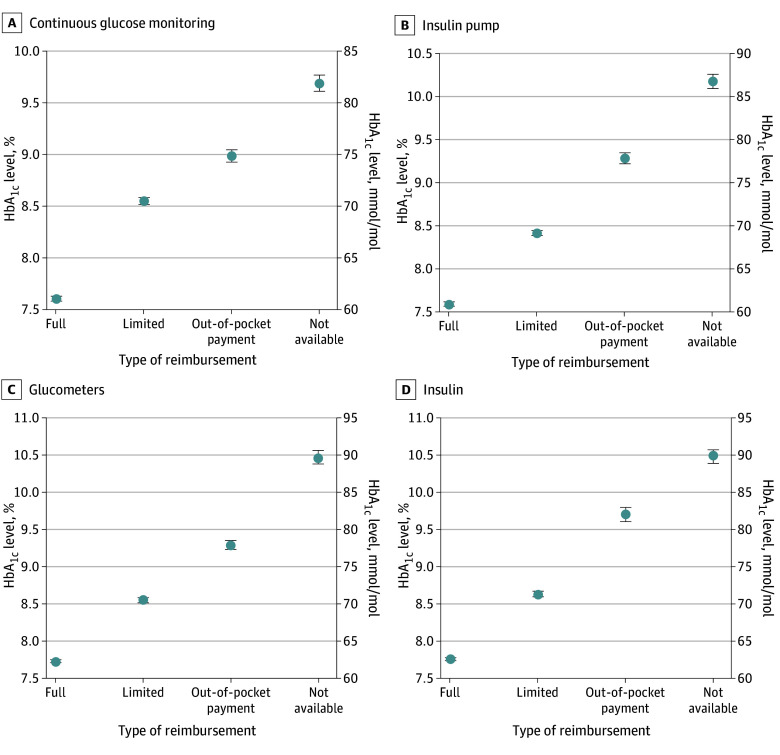
Glycated Hemoglobin (HbA_1c_) Level by Type of Reimbursement for Diabetes Technology HbA_1c_ levels are presented as means; whiskers represent 95% CIs. The lowest HbA_1c_ level was reached by children with type 1 diabetes treated in centers with full reimbursement of all the technologies. The differences between the types of reimbursement are statistically significant for all technologies (*P* < .001).

Significant and consistent differences in HbA_1c_ levels were observed among the 4 categories of reimbursement in all technologies (Table and Figure 2). The lowest values of HbA_1c_ were observed in centers with full reimbursement of the given technology. This result was seen for CGM (7.62% [95% CI, 7.59%-7.64%]; 59.8 [59.4-60.0] mmol/mol), as well as CSII (7.61% [95% CI, 7.59%-7.64%]; 59.7 [59.5-60.0] mmol/mol), glucometers (7.73% [95% CI, 7.71%-7.75%]; 61.0 [60.8-61.2] mmol/mol), and insulin (7.75% [95% CI, 7.73%-7.77%]; 61.2 [61.0-61.4] mmol/mol). Conversely, the highest HbA_1c_ levels were recorded in the centers where the given technology was not available (9.65% [95% CI, 9.55%-9.71%]; 82.0 [80.1-82.6] mmol/mol for CGM and 10.10% [95% CI, 10.01%-10.19%]; 86.9 [85.9-87.9] mmol/mol for CSII) or was provided through sponsorship (10.49% [95% CI, 10.40%-10.58]; 91.2 [90.2-92.1] mmol/mol for glucometers and 10.49% [95% CI, 10.40%-10.58%]; 91.2 [90.2-92.1] mmol/mol for insulin), followed by the centers with out-of-pocket availability (8.97% [95% CI, 8.91%-9.03%]; 74.5 [73.9-75.2] mmol/mol for CGM; 9.31% [95% CI, 9.24%-9.37%]; 78.3 [77.5-78.9] mmol/mol for CSII; 9.24% [95% CI, 9.18%-9.30%]; 77.5 [76.8-78.1] mmol/mol for glucometers; 9.72% [95% CI, 9.62%-9.82%]; 82.7 [81.6-83.8] mmol/mol for insulins) and the centers with limited reimbursement (8.57% [95% CI, 8.54%-8.60%]; 70.2 [69.8-70.5] mmol/mol] for CGM; 8.42% [95% CI, 8.39%-8.44%]; 68.6 [68.2-68.7] mmol/mol for CSII; 8.59% [95% CI, 8.55%-8.63%]; 70.4 [70.0-70.8] mmol/mol for glucometers; and 8.65% [95% CI, 8.62%-8.69%]; 71.0 [70.7-71.5] mmol/mol for insulin).

**Table.** HbA_1c_Values Achieved by Children With Type 1 Diabetes in All Reimbursement Categories for Each Technology and Insulin

Technology and insulin	HbA_1c_ level, mean (95% CI), %^a^				*P* value
	Full availability and reimbursement	Limited reimbursement	Out-of-pocket payment	Available by sponsor or not available	
CGM	7.62 (7.59-7.64) [59.8 (59.4-60.0)]	8.57 (8.54-8.60) [70.2 (69.8-70.5)]	8.97 (8.91-9.03) [74.5 (73.9-75.2)]	9.65 (9.55-9.71) [82.0 (80.1-82.6)]	<.001
CSII	7.61 (7.59-7.64) [59.7 (59.5-60.0)]	8.42 (8.39-8.44) [68.6 (68.2-68.7)]	9.31 (9.24-9.37) [78.3 (77.5-78.9)]	10.10 (10.01-10.19) [86.9 (85.9-87.9)]	<.001
Glucometers	7.73 (7.71-7.75) [61.0 (60.8-61.2)]	8.59 (8.55-8.63) [70.4 (70.0-70.8)]	9.24 (9.18-9.30) [77.5 (76.8-78.1)]	10.49 (10.40-10.58) [91.2 (90.2-92.1)]	<.001
Insulin	7.75 (7.73-7.77) [61.2 (61.0-61.4)]	8.65 (8.62-8.69) [71.0 (70.0-71.5)]	9.72 (9.62-9.82) [82.7 (81.6-83.8)]	10.49 (10.40-10.58) [91.2 (90.2-92.1)]	<.001

Abbreviations: CGM, continuous glucose monitoring; CSII, continuous subcutaneous insulin infusion (insulin pump); HbA_1c_, glycated hemoglobin.

^a^ The lowest values of HbA_1c_ are associated with full availability and reimbursement. This could be seen for each technology. Data in brackets are values given as mmol/mol.

**Figure 3.**
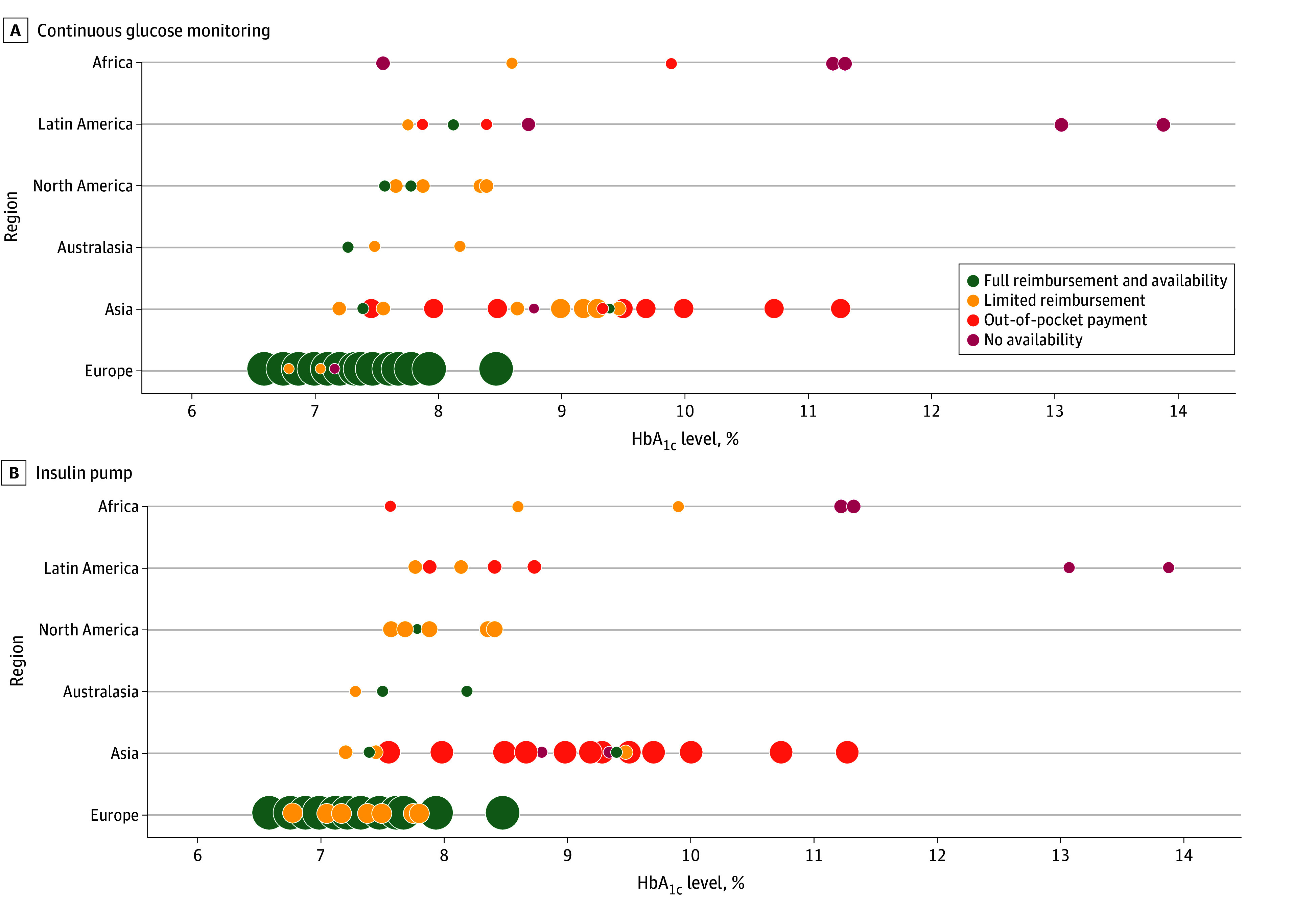
The Distribution of Individual Centers From Different Continents by Glycated Hemoglobin (HbA_1c_) Value and Type of Reimbursement Bubble size reflects the number of centers that fall into the same HbA_1c_ range (within 1%) and reimbursement category within a specific continent. The smallest bubble size represents a center that is alone in its category, whereas the largest bubble size indicates that more than 10 centers share the same category.

The distribution of individual centers by glycemic outcomes and continents and the type of reimbursement of CGM and CSII are shown in Figure 3. Centers with full reimbursement of these technologies achieved the lowest HbA_1c_ levels, but there were overlaps between categories in individual centers.

Consequently, the target of HbA_1c_ levels of less than 6.5% (<48 mmol/mol)^17^ was reached by 18.7% and 19.1% of children with T1D with full accessibility to CGM and CSII, respectively. This represents a significant difference compared with centers with limited reimbursement (9.2% and 10.5%, respectively), out-of-pocket payment (8.4% and 5.0%, respectively), and no availability of these technologies (7.8% and 5.1%, respectively) (eFigure 1 in Supplement 1).

## Discussion

This study reveals substantial disparities in the accessibility and reimbursement of diabetes technologies and insulin for children with T1D, depending on their continent and country of residence. These differences are closely linked to variations in glycemic outcomes. Full reimbursement of technologies is associated with the lowest HbA_1c_ levels. As the most globally representative data to date, the findings highlight the substantial efforts required to enhance access and improve equity in glycemic outcomes.

For recent years, our data reflect improved access and reimbursement in some regions. For example, the observation for Europe is that in 2009 only Sweden and Slovenia offered full reimbursement for a CGM.^15^ By 2017, there was widespread access (with notable exemptions in Croatia, Latvia, Portugal, and Romania).^6^ As of 2024, there was full coverage for CGM in Europe (except for Montenegro, where technology was not sufficiently accessible, and Poland, where some limitations in terms of copayments were required). Nevertheless, in 2024 disparities within Europe were still evident. For example in Bulgaria, Latvia, and Montenegro, CSII itself was fully covered, but HCL technology remained unavailable.

Outside Europe, reimbursement policies differed substantially, resulting in much poorer levels of glycemic outcomes. In some parts of North America, some technologies were partially reimbursed due to significant copayments depending on an individual type of health insurance and strict indication criteria. Importantly, this inequality applied not only to technologies but also to insulin. Moreover, accessibility and reimbursement differed across states and provinces. Heterogeneous reimbursement status was also reported in Latin America or Asia, where reimbursement within a country ranged from full coverage for CGM or CSII to no coverage in Brazil, Bolivia, or India. Alarmingly, some countries reported insufficient access to insulin alone; for example in Africa, despite a small number of participating countries (n = 6), 3 (50%) reported inadequate access to both technologies and insulin.

There is substantial evidence linking the use of modern technologies not only with improved quality and flexibility of life^18,19^ but also with significant benefits for glycemia.^20,21,22,23^ However, limited access to diabetes technologies due to financial constraints remains a major barrier to their widespread use,^24^ which can ultimately prevent individuals from achieving recommended glycemic targets. The significance of permanent technology use is supported by a recent study^25^ showing that CGM use for 90% or greater is associated with healthier glycemia compared with use of 70% to 89%. The importance of reimbursement is also highlighted by another study on children with T1D^26^ confirming that introduction of national reimbursement for CGM led to a significant reduction in HbA_1c_ levels. Similar findings were reported in a recent longitudinal analysis of data from 9 pediatric diabetes registries^9^ and were also observed in Ukrainian refugee children with T1D who experienced improved glycemic outcomes after the initiation of CGM without any financial burden.^27^ Additionally, data from German-Austrian and US registries comparing CGM use for several years showed variations in CGM adoption rates across pediatric and adult populations, which can likely be attributed to differences in reimbursement policies.^28^ These findings align with our data, which show that children with T1D in countries with full reimbursement of diabetes technologies tend to achieve the lowest HbA_1c_ levels. Therefore, it is essential to explore solutions that ensure full access to these technologies for children with T1D. Making these technologies more accessible should be a priority for both government leaders and technology companies. In addition, there is an urgent need to improve interoperability between devices. Simplified compatibility between systems can reduce costs and improve results by facilitating the seamless integration of data and tools for optimal diabetes management.^29^

### Strengths and Limitations 

The main strengths of our study are the large number of countries participating in this survey and the unique possibility of linking the reimbursement data with glycemic outcomes. Moreover, this study was performed within the SWEET project, which enables a unique global network, standardized data collection, and benchmarking capabilities and so has been instrumental in displaying the impact of disparities in diabetes outcomes of children with T1D worldwide in such a large picture.

Although the data presented herein are unambiguous, we acknowledge the limitations of our data with respect to other factors (educational, ethnic and cultural, nutritional, etc) that may affect glycemic outcomes. Moreover, adequate staffing of diabetes clinics, their standard organizational structure including the presence of nonmedical members of diabetes centers,^29^ and a sufficient network of specialists within the country contribute to the outcomes.^30,31^ This concept is in line with a significant overlap in HbA_1c_ levels among the 4 reimbursement categories (Figure 3). A further limitation is that the analyses were performed on data collected through questionnaires, and therefore we cannot prove causality. Moreover, some regions are only represented by a small number of centers and may not be representative of the whole country. Additionally, this study did not focus on quality-of-life outcomes that could add value to our work, as this could also be associated with the accessibility of insulin and technologies.

## Conclusions

In this cross-sectional study of the association of accessibility and reimbursement with glycemic outcomes, we observed that HbA_1c_ levels were associated with the accessibility of modern diabetes technologies and insulin. We conclude that the greatest challenge to achieving global equity in diabetes outcomes lies in the unequal access to modern technologies for all children with T1D. While some countries advance toward comprehensive diabetes management, others still struggle with basic access to life-preserving insulin. This stark disparity underscores the urgent need for collective action. These data serve as a call to accelerate ongoing initiatives and inspire new, innovative solutions aimed at closing these gaps. Only by addressing these inequities can we ensure that every child with diabetes, regardless of their geographic or socioeconomic status, has the same opportunity in diabetes care and diabetes outcomes.

## Notes

Supplement 1. **eTable 1.** Mean HbA_1c_ Values Achieved by Children With T1D in All Reimbursement Categories for Each Technology and Insulin (2023 Data Only) **eTable 2.** Basic Characteristics of the Study Group **eTable 3.** The List of Participating Countries and the Type of Reimbursement in Individual Countries **eFigure.** The Percentage of Children With T1D Achieving the HbA_1c_ Target <48 mmol/mol (6.5%) in Association With Technology Reimbursement **eAppendix 1.** Study Questionnaire **eAppendix 2.** List of Participating Centers

Supplement 2.
Data Sharing Statement
