# Supplementary material for: Global Inequities in Diabetes Technology and Insulin Access and Glycemic Outcomes
Source: JAMA Netw Open. 2025 Aug 27;8(8):e2528933. doi: 10.1001/jamanetworkopen.2025.28933 (PMC12391998; doi:10.1001/jamanetworkopen.2025.28933)
Supplement: Supplement 1. — eTable 1. Mean HbA1c Values Achieved by Children With T1D in All Reimbursement Categories for Each Technology and Insulin (2023 Data Only) eTable 2. Basic Characteristics of the Study Group eTable 3. The List of Participating Countries and the Type of Reimbursement in Individual Countries eFigure. The Percentage of Children With T1D Achieving the HbA1c Target <48 mmol/mol (6.5%) in Association With Technology Reimbursement eAppendix 1. Study Questionnaire eAppendix 2. List of Participating Centers [file jamanetwopen-e2528933-s001.pdf]

Supplementary Online Content

Santova A, de Bock M, Lanzinger S, et al; for the SWEET Study Group. Global inequities in diabetes technology and insulin access and glycemic outcomes. *JAMA Netw Open.* 2025;8(8):e2528933. doi:10.1001/jamanetworkopen.2025.28933

eTable 1. Mean HbA_{1c} Values Achieved by Children With T1D in All Reimbursement Categories for Each Technology and Insulin (2023 Data Only)
eTable 2. Basic Characteristics of the Study Group
eTable 3. The List of Participating Countries and the Type of Reimbursement in Individual Countries
eFigure. The Percentage of Children With T1D Achieving the HbA_{1c} Target <48 mmol/mol (6.5%) in Association With Technology Reimbursement
eAppendix 1. Study Questionnaire
eAppendix 2. List of Participating Centers

This supplementary material has been provided by the authors to give readers additional information about their work.

© 2025 Santova A et al. *JAMA Network Open*.

eTable 1. Mean HbA_{1c} Values Achieved by Children With T1D in All Reimbursement Categories for Each Technology and Insulin (2023 Data Only)

	Mean HbA_{1c} Values (% and mmol/mol, 95% CI)				
	Full availability and reimbursement	Limited reimbursement	Out-of-pocket payment	Available by sponsor/Not available	*P*-value
CGM	7.48 [7.46-7.51] 58.3 [58.0-58.6]	8.29 [8.26-8.33] 67.1 [66.8-67.6]	8.67 [8.59-8.74] 71.3 [70.4-72.0]	9.43 [9.34-9.52] 79.6 [78.6-80.6]	<0.001
CSII	7.49 [7.46-7.52] 58.4 [58.0-58.7]	8.17 [8.14-8.20] 65.8 [65.5-66.1]	9.25 [9.16-9.34] 77.6 [76.6-78.6]	9.88 [9.78-9.98] 84.5 [83.4-85.6]	<0.001
Glucometers	7.58 [7.56-7.61] 59.3 [59.1-59.7]	8.28 [8.24-8.32] 67.0 [66.6-67.4]	9.19 [9.11-9.26] 76.9 [76.1-77.7]	10.25 [10.14-10.37] 88.5 [87.3-89.8]	<0.001
Insulins	7.63 [7.61-7.66] 59.9 [59.7-60.2]	8.39 [8.35-8.43] 68.2 [67.8-68.6]	9.85 [9.69-10.01] 84.2 [82.4-85.9]	10.29 [10.18-10.41] 89.0 [87.7-90.3]	<0.001

© 2025 Santova A et al. *JAMA Network Open*.

eTable 2 . Basic Characteristics of the Study Group

T1D – type 1 diabetes

	Europe	North America	Latin America	Asia	Africa	Australasia	Total N = 42349
**Centers N (%)**	39 (48%)	6 (7.4%)	8 (9.9%)	19 (24%)	6 (7.4%)	3 (3.7%)	81
**Children with diabetes N (%)**	21943 (52%)	10042 (24%)	1570 (3.7%)	4735 (11%)	2826 (6.7%)	1233 (2.9%)	42349
**Gender – male (%)**	53%	53%	49%	49%	50%	51%	52%
**Age [years] mean (SD)**	14 (4.3)	15 (4.4)	13 (4.1)	14 (4.6)	14 (4.8)	13 (3.6)	14 (4.4)
**T1D duration [years] mean (SD)**	6.1 (4.2)	6.5 (4.3)	4.8 (3.3)	5.6 (3.9)	5.1 (3.8)	5.3 (3.7)	6.0 (4.2)
**Insulin dose [U/kgBW/day] mean (SD)**	0.79 (0.29)	0.85 (0.28)	0.91 (0.34)	0.99 (0.37)	0.88 (0.31)	0.84 (0.22)	0.84 (0.31)
**BMI-SDS (mean (SD))**	0.56 (1.0)	0.82 (1.2)	0.54 (1.2)	-0.06 (1.4)	-0.07 (1.2)	0.90 (1.1)	0.52 (1.2)

© 2025 Santova A et al. *JAMA Network Open*.

eTable 3. The List of Participating Countries and the Type of Reimbursement in Individual Countries

Canada and the USA are divided into provinces that provided responses. The countries marked with an asterisk (*) also responded for the part of the country (center/province/territorial unit).
1 = Full availability and reimbursement 2 = Limited reimbursement 3 = Out-of-pocket payment 4 = Not available (for CGM and CSII) or Supported by sponsors

	Country	CGM availability	CSII availability	Glucometers availability	Insulin availability
EUROPE	Austria	1	1	1	1
	Belgium	1	1	1	1
	Bulgaria	1	2	1	1
	Croatia	1	2	1	1
	Czech Republic	1	1	1	1
	Denmark	1	1	1	1
	France	1	1	1	1
	Germany	1	1	1	1
	Greece	1	1	1	1
	Hungary	1	1	1	1
	Italy*	1	1	1	1
	Ireland	1	1	1	1
	Latvia	1	2	1	1
	Lithuania	1	2	1	1
	Luxembourg	1	1	1	1
	Montenegro	4	2	1	1
	Norway	1	1	1	1
	Poland	2	2	1	1
	Portugal	1	1	1	1
	Romania	1	1	2	1
	Slovenia	1	1	1	1
	Switzerland	1	2	1	1
	Spain	1	1	1	1
	Sweden	1	1	1	1
	The Netherlands	1	1	1	1
	UK*	1	1	1	1
NORTH AMERICA	Canada (Alberta)	1	1	1	2
	Canada (Nova Scotia)	2	2	1	2
	Canada (Ontario)	1	2	1	1
	Canada (Quebec)	1	1	1	1
	USA (Colorado)	2	2	2	2
	USA (Massachusetts)	2	2	2	2
LATIN AMERICA	Argentina*	2	2	1	1
	Brazil	3	3	2	2
	Bolivia	3	3	4	4
	Chile	2	2	1	1
	Costa Rica	1	2	1	1
	Ecuador	4	3	3	2
	Haiti	4	4	4	4
	Peru	2	3	3	1

© 2025 Santova A et al. *JAMA Network Open*.

ASIA	Hong Kong*	2	3	3	1
	India	3	3	3	3
	Iran	4	4	3	2
	Israel	1	1	1	1
	Kuwait	1	1	3	1
	Maldives	2	2	4	1
	Nepal	4	4	4	4
	Pakistan	3	3	4	4
	South Korea	2	2	2	2
	Taiwan	2	3	1	1
	Thailand	2	3	2	1
	Turkiye	3	2	2	1
	Egypt	3	2	1	1
	Ghana*	4	4	4	4
AFRICA	Mali	4	4	4	4
	Mauritius	4	3	1	1
	Morocco*	2	2	1	1
	Senegal	4	4	4	4
AUSTRALASIA	Australia	1	3	1	1
	New Zealand	3	1	1	1

© 2025 Santova A et al. *JAMA Network Open*.

eFigure. The Percentage of Children With T1D Achieving the HbA_{1c} Target <48 mmol/mol (6.5%) in Association With Technology Reimbursement

CGM (A) and CSII (B). The whiskers represent 95%-confidence intervals.

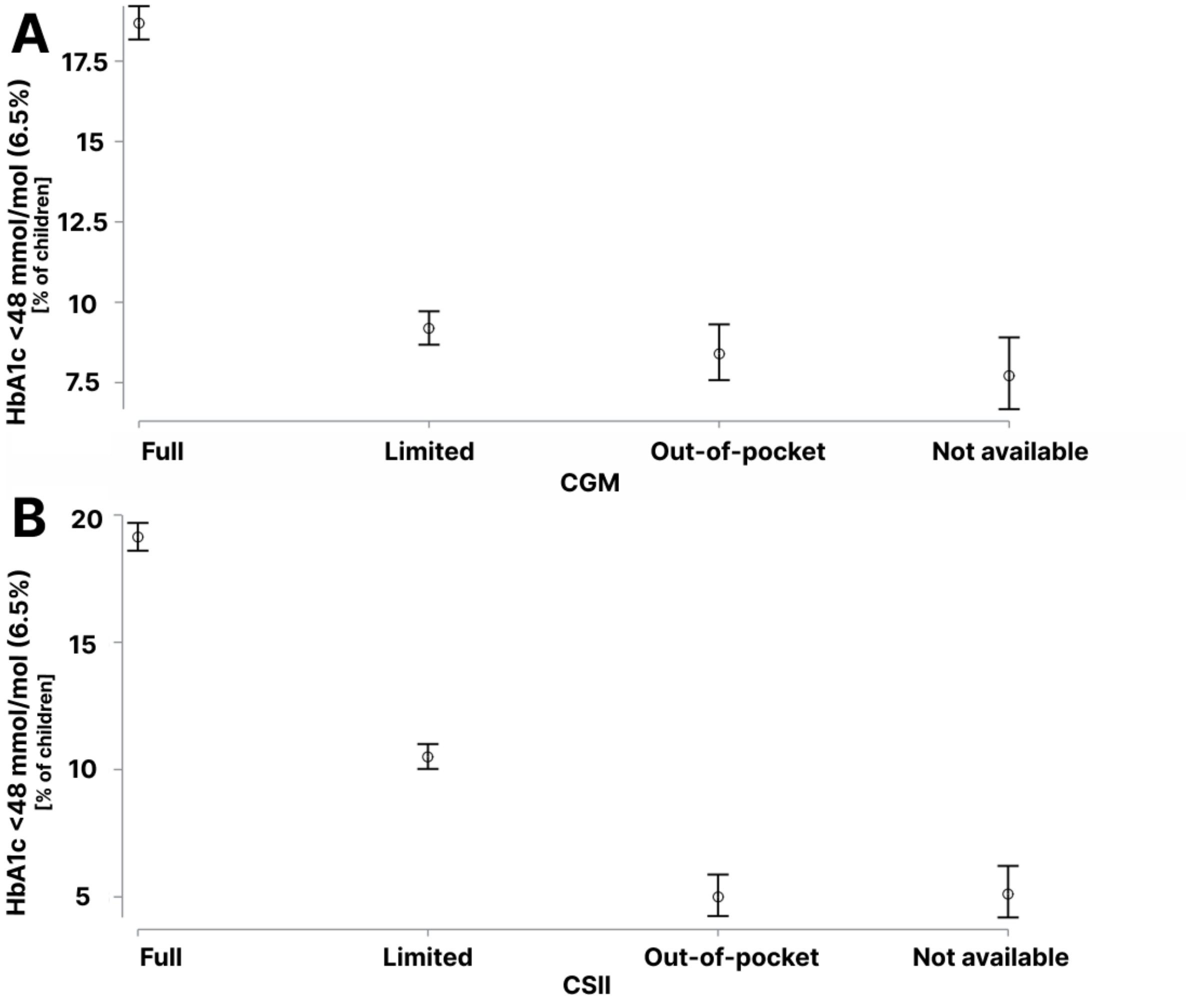

© 2025 Santova A et al. *JAMA Network Open*.

eAppendix 1. Study Questionnaire

CGM

Section 2 – CGM availability

2.1. IS CGM technology available in your country/province/state? *

a) Yes (please continue to Section 3 - CGM)

b) No (please skip to Section 6 – Glucometers)

Section 3 – CGM

2.2. How are CGMs in your country/province/state covered for the majority of children with type 1 diabetes? *

a) Full reimbursement (fully covered by government/employers, allowing >90% of CGM use per year) (please skip to Section 5 after filling this section)

b) Limited reimbursement (co-payment needed/ geographical/age-dependent/insurance-dependent differences) (please continue to Section 4 after filling this section)

c) Out-of-pocket (no reimbursement in the country/province/state, it is possible to purchase the device independently) (please skip to Section 6 (Glucometers) after filling this section)

d) Private insurance/prepaid medicine (please skip to Section 6 (Glucometers) after filling this section)

e) By sponsors (the technology is not fully funded by government/employer in the country/state/province, but unrestricted access to the technology is ensured through foundations or other similar sources) (please skip to Section 6 (Glucometers) after filling this section)

f) Not able to estimate (please skip to Section 6 (Glucometers) after filling this section)

2.3. Please estimate for the country/province/state: What is the estimated proportion (%) of children and adolescents with T1D using CGM whose CGM is covered by (calculated as percentage of CGM users): * (for each line a-d choose one of the options – from 0-100%/or not able to estimate)

a) Full reimbursement (allowing >90% of CGM use per year)

b) Limited reimbursement

c) Out-of-pocket

d) Private insurance/prepaid medicine

e) Sponsors

2.4. What is the estimated proportion (%) of children and adolescents with T1D **at your center** using a CGM for each percentage of time: * (for each line a-d choose one of the options – from 0-100%)

a) >90% of time

b) 70-90% of time

c) 30-70% of time

d) <30% of time

Section 4 – CGM Limited coverage (please skip to Section 6 - Glucometers after filling this section)

2.5. Which of the following limitations for prescribing CGM in children with T1D are relevant for your country/province/state? * (multiple choice question)

a) Reimbursement depends on the insurance type

b) Age-dependent differences

c) Geographical differences

d) Some co-payments are needed

e) Other

2.6. Describe each of the limitations in more detail* ________________

2.7. Are there any indication criteria for CGM prescription for children and adolescents that significantly limit access to these technologies? *

a) Yes

b) No – the only indication is the diagnosis of T1D itself

© 2025 Santova A et al. *JAMA Network Open*.

2.8. If yes, describe the indications briefly: ____________________

Section 5 – CGM Full reimbursement (please continue to Section 6 (Glucometers) after filling this section)

2.9. Are there any indication criteria for CGM prescription that significantly limit access to these technologies? *

a) Yes
b) No – the only indication is the diagnosis of T1D itself

2.10. If yes, describe the indications briefly: ____________________

GLUCOMETERS

Section 6 – Glucometers

3.1. How are glucometers and glucometer strips in your country/province/state covered for the majority of children with type 1 diabetes? *

a) Full reimbursement (fully covered by government/employers, allowing glucose level measurement >5 times daily) (please skip to Section 8 (CSII availability) after filling this section)
b) Limited reimbursement (some co-payment needed/there are some geographical/age-dependent/insurance-dependent differences within the country/province/state) (please continue to Section 7 after filling this section)
c) Out-of-pocket (no reimbursement in the country/province/state, it is possible to purchase the device independently) (please skip to Section 8 (CSII availability) after filling this section)
d) Private insurance/prepaid medicine (please skip to Section 6 (Glucometers) after filling this section)
e) By sponsors (the technology is not fully funded by government/employer in the country/state/province, but unrestricted access to the technology is ensured through foundations or other similar sources) (please skip to Section 8 (CSII availability) after filling this section)
f) Not able to estimate (please skip to Section 8 (CSII availability) after filling this section)

3.2. Please estimate for the country/province/state: What is the estimated proportion (%) of children and adolescents with T1D using glucometers whose glucometer and glucometer strips are covered by (calculated as percentage of glucometer users): * (for each line a-d choose one of the options – from 0-100%/or not able to estimate)

a) Full support reimbursement (allowing glucose level measurement >5 times daily)
b) Limited reimbursement
c) Out-of-pocket
d) Private insurance/prepaid medicine
e) Sponsors

3.3. What is the estimated proportion (%) of children and adolescents with T1D monitored EXCLUSIVELY by glucometers (without CGM monitoring) **at your center**? * ______________

3.4. What is the estimated proportion (%) of children and adolescents with T1D using EXCLUSIVELY a glucometer who monitor blood glucose level using a glucometer (please estimate for the center): * (for each line a-d choose one of the options – from 0-100%/not able to estimate)

a) Once daily
b) 2-3x daily
c) More than three times daily
d) Do not monitor their blood glucose level

Section 7 – Glucometers Limited coverage (please skip to Section 8 (CSII availability) after filling this section)

3.5. Which of the following limitations for prescribing glucometers in children with T1D are relevant for your country/province/state? * (multiple choice question)

a) Reimbursement depends on the insurance type
b) Age-dependent differences
c) Geographical differences
d) Some co-payments are needed
e) Limited number of reimbursed glucometer strips yearly

f) Other

3.6. Describe each of selected limitations in more detail* ________________

INSULIN PUMPS - CSII

Section 8 – CSII availability

4.1. Are insulin pumps (CSII) available in your country/province/state? *

a) Yes, including HCL (hybrid closed loop) (please continue to Section 9 - CSII)
b) Yes, without HCL systems (please continue to Section 9 - CSII)
c) No (please skip to Section 12 - Insulins)

Section 9 – CSII

4.2. How are CSII in your country/province/state covered for the majority of children with type 1 diabetes? *

a) Full reimbursement including pump supplies (fully covered by government/employer) (please skip to Section 11 after filling this section)
b) Limited reimbursement (some co-payment needed/there are some geographical/age-dependent/insurance-dependent differences within the country/province/state) (please continue to Section 10 after filling this section)
c) Out-of-pocket (no reimbursement in the country/province/state, it is possible to purchase the device independently) (please skip to Section 12 (Insulins) after filling this section)
d) Private insurance/prepaid medicine (please skip to Section 6 (Glucometers) after filling this section)
e) By sponsors (the technology is not fully funded by government/employer in the country/state/province, but unrestricted access to the technology is ensured through foundations or other similar sources) (please skip to Section 12 (Insulins) after filling this section)
f) Not able to estimate

4.3. Please estimate for the country/province/state: What is the estimated proportion (%) of children and adolescents with T1D using CSII whose CSII is covered by (calculated as percentage of CSII users): * (for each line a-d choose one of the options – from 0-100%/or no table to estimate)

a) Full reimbursement
b) Limited reimbursement
c) Out-of-pocket
d) Private insurance/prepaid medicine
e) Sponsors

4.4. What is the estimated proportion (%) of children and adolescents with T1D using CSII (with or without automated function) **at your center?** * ____________

4.5. What is the estimated proportion (%) of children and adolescents with T1D using CSII with HCL algorithm (if available) **at your center?** (calculated as a percentage of children who are using insulin pumps) __________

Section 10 – CSII Limited coverage (after filling this section continue to Section 12 – Insulins)

4.6. Which of the following limitations for prescribing CSII in children with T1D are relevant for your country/province/state? * (multiple choice question)

a) Reimbursement depends on the insurance type
b) Age-dependent differences
c) Geographical differences
d) Some co-payments are needed
e) No reimbursement of HCLs
f) Other

4.7. Describe each of the limitations in more detail including limitations for CSII supplies (and including HCL technology, if available) * ________________

© 2025 Santova A et al. *JAMA Network Open*.

4.8. Are there any <u>indication criteria</u> for CSII prescription for children and adolescents (including HCL, if available) that significantly limit access to these technologies *

a) Yes
b) No – the only indication is the diagnosis of T1D itself

4.9. If yes, describe the indications briefly: ____________________

<u>Section 11 – CSII Full reimbursement</u> (please continue to Section 12 (Insulins) after filling this section)

4.10. Are there any <u>indication criteria</u> for CSII prescription for children and adolescents (including HCL, if available) that significantly limit access to these technologies *

a) Yes
b) No – the only indication is the diagnosis of T1D itself

4.11. If yes, describe the indications briefly: ____________________

4.12. Are there any limitations for the prescription of CSII supplies? *

a) Yes
b) No

4.13. If yes, describe briefly _______________

INSULINS

<u>Section 12 – Insulins</u>

5.1. How are insulins in your country/province/state covered for the majority of children with type 1 diabetes? *

a) Full reimbursement (fully covered by government/employer) (please skip to Section 14 (Diabetes care organization) after filling this section)
b) Limited reimbursement (some co-payment needed/there are some geographical/age-dependent/insurance-dependent differences within the country/province/state or no analogs reimbursed) (please continue to Section 13 after filling this section)
c) Out-of-pocket (no reimbursement in the country/province/state, it is possible to purchase insulins independently) (please skip to Section 14 (Diabetes care organization) after filling this section)
d) Private insurance/prepaid medicine (please skip to Section 6 (Glucometers) after filling this section)
e) By sponsors (insulin is not fully funded by government/employer in the country/state/province, but unrestricted access to it is ensured through foundations or other similar sources) (please skip to Section 14 (Diabetes care organization) after filling this section)
f) Not able to estimate

5.2. What is the estimated proportion (%) of children and adolescents with T1D whose insulin is in your country/province/state covered by (please estimate for the country/province/state): * (for each line a-d choose one of the options – from 0-100%/or no table to estimate)

a) Full reimbursement
b) Limited reimbursement
c) Out-of-pocket
d) Private insurance/prepaid medicine
e) Sponsors

5.3. Estimate the proportion (%) of children and adolescents with T1D **at your center** treated with: * (for each line a-c choose one of the options – from 0-100%/or no table to estimate)

a) Human insulins (without analogs)
b) Human insulins combined with analogs
c) Insulin analogs only

<u>Section 13 – Insulins Limited coverage</u> (please continue to Section 14 – Diabetes care organization)

5.4. Which of the following limitations for prescribing insulins in children with T1D are relevant for your country/province/state? * (multiple choice question)

© 2025 Santova A et al. *JAMA Network Open*.

a) Reimbursement depends on the insurance type
b) Age-dependent differences
c) Geographical differences
d) Some co-payments are needed
e) Some types of insulins are not covered (f.e. insulin analogs)
f) Other

5.5. Describe each of the limitations in more detail * ________________

DIABETES CARE ORGANIZATION

Section 14 – Diabetes care organization

6.1. What is the estimated proportion (%) of children and adolescents with T1D who are followed up by: (please estimate for the country/province/state): * (for each line a-d choose one of the options – from 0-100%/or no table to estimate)

a) Pediatric diabetologist (at a diabetes center)
b) Pediatric diabetologist (not at a diabetes center)
c) Diabetologist for adult
d) Pediatrician
e) General practitioner
f) Other

CONCLUSION

Section 15 – Additional notes

7.1. Place for additional notes if needed ___________

© 2025 Santova A et al. *JAMA Network Open*.

eAppendix 2. List of Participating Centers

COUNTRY	NAME	City	Center name
**LATIN AND NORTH AMERICA**			
Argentina	Consuelo Barcala	Buenos Aires	Unit of Nutrition Hospital Juan P. Garrahan
Brazil	Mauro Scharf Pinto	Curitiba-Parana	Centro de Diabetes Curitiba
Canada	Beth Cummings	Halifax	IWK Health Centre
Canada	Alanna Landry	Markham	Markham Stouffville Hospital
Canada	Ellen Goldbloom	Ottawa	Children's Hospital of Eastern Ontario
Canada	Laurent Legault	Montreal	Montreal Children’s Hospital/McGill University Health Center
Canada	Daniele Pacaud	Calgary	Alberta Children’s Hospital
Chile	Julie Pelicand	San Felipe	Hospital San Camilo
Chile	Franco Giraudo	Santiago	Maternal and Child Research Institute, University of Chile
Cost Rica	Erick Richmond	San José	National Children's Hospital, Hospital CIMA
Ecuador	Ana Fernanda Sanchez Encalada	Cuenca	Fundación Los Fresnos "Casa de la Diabetes"
Haiti	Alexandra Mayard	Port-au-Prince	Fondacion Hatienne de Diabete et de Maladies Cardio-Vasc.
Haiti	Martine Canal	Montrouis	Kay Mackenson
Peru	Segundo Seclen	Lima	Asociacion de Diabeticos Juveniles
USA	Guy Todd Alonso	Denver	Barbara Davis Center for Diabetes
USA	Katharine Garvey	Boston	Boston Children's Hospital
**AFRICA**			
Egypt	Nancy Elbarbary	Cairo	Ain Shams University, Faculty of Medicine
Ghana	Emmanuel Ameyaw	Kumasi	Komfo Anokye Teaching Hospital
Mali	Stephane Besancon	Bamako	NGO Santé Diabète
Morocoo	Zineb Imane	Rabat	Children’s Hospital – Unit Of Pediatric Diabetology
Senegal	Djibril Boiro	Dakar	Centre Hospitalier Abass Ndao
**EUROPE**			
Austria	Birgit Rami	Vienna	Universitätsklinik für Kinder- und Jugendheilkunde
Belgium	Kristina Cateels	Leuven	University Hospital Leuven
Bulgaria	Violeta Iotova	Varna	University Hospital „Sv. Marina“
Croatia	Marija Pozgaj	Zagreb	University Clinical Hospital Center "Sestre milosrdnice"
Croatia	Nevena Krnich	Zagreb	University Hospital Center Zagreb
Czechia	Zdeněk Šumník	Prague	Motol University Hospital

© 2025 Santova A et al. *JAMA Network Open*.

Denmark	Jannet Svensson	Herlev	Steno Diabetes Center Copenhagen
Denmark	Niels Birkebaek	Aarhus	Skejby Hospital, University of Århus
France	Julie Pelicand	Toulouse	University Hospital Center of Toulouse
Germany	Torben Biester	Hannover	Kinderkrankenhaus auf der Bult
Greece	Ioanna Kosteria	Athens	Dep. of Endocrinology, Growth & Development at P&A Kyriakou
Greece	Christina Kanaka-Gantenbein	Athens	Agia Sophia" Children's Hospital
Greece	Maria Xatzipsalti		P&A Kyriakou Children's Hospital
Hungary	Peter Toth-Heyn	Budapest	Semmelweis University
Italy	Bonfanti Riccardo	Milan	Ospedale San Raffaele
Italy	Claudio Maffeis	Verona	U.O.C. di Pediatria
Italy	Barbara Piccini	Florence	Meyer Children's Hospital
Italy	Luisa Desanctis	Turin	Centro Diabetologia Pediatrica
Italy	Enza Mozzilo	Naples	University of Naples
Ireland	Conor Cronin	Cork	Cork University Hospital
Latvia	Iveta Dzivite	Riga	Children's University Hospital
Lithuania	Romualdas Tomas Preiksa	Kaunas	Endocrinolgoy of Hospital of LUHS Kauno
Luxembourg	Michael Witsch	Luxembourg	Pediatric Clinic CH de Luxembourg
Montenegro	Maja Racievic	Podgorica	Insitute for children's diseases, Clinical center of Montenegro
Norway	Heiko Bratke	Haugesund	Department of Pediatrics, Haugesund, Helse Fonna
Poland	Agata Chobot	Opole	Department of Pediatrics, University of Opole
Poland	Agnieszka Szadowska, Beata Miankowska	Lodz	Medical University of Lodz
Portugal	Catarina Limbert	Lisbon	Hospital Dona Estefânia Lisbon
Portugal	Sofia Castro	Lisbon	Associação Protectora dos Diabéticos de Portugal
Romania	Mihaela Victoria Vlaiculescu	Bucharest	CLINICA DIABNUTRIMED
Romania	Gabriela Florian	Baia Mare	Spitalul Judetean de Urgenta "Dr. Constantin Opris"
Slovenia	Natasa Bratina	Ljubljana	University Children's Hospital
Switzerland	Marie Anne Burckhardt	Basel	University Children Hospital Basel
Spain	Roque Cardona	Barcelona	Hospital Sant Joan de Deu
Spain	Luis Castano	Barakaldo-Bilbao	Hospital Universitario Cruces
Spain	Mireia Tirado Capistros	Barcelona	Hospital de la Santa Creu i Sant Pau
Spain	Cristina Aguilar	Barcelona	Hospital Maternoinfantil Vall Hebron

© 2025 Santova A et al. *JAMA Network Open*.

Sweden	Auste Pundziute Lycka	Gothenborg	Queen Silvia Childrens Hospital
The Netherlands	Theo Sas	Rotterdam	Diabeter Nederland
UK	Ursula Ngwu	Mansfield	Sherwood Forest Hospital NHS
**ASIA**			
China	Joanna Yuet-ling Tung	Hong Kong	Hong Kong Childrens Hospital
India	G D Ramchandani	Kota	Ramchandani Diabetes Care and Research Centre
India	Banshi Saboo	Ahmedabad	Diacare Hormone Cinic
India	Sujata Jali	Belgaum	KLES Diabetes centre . Dr Prabhakar Kore Hospital and MRC Belgaum
India	Gopika Beena Chandran	India	Jothyden Kesavavdev, Gopika Beena Chandran
India	Rutul Gokalani	Ahmedabad	Arogyam Health Care (AHC Diabetes Clinic)
India	Yash Patel	Ahmedabad	SWASTHYA DIABETES CARE
India	Leela Baid	Chennai	M. V. Hospital for Diabetes and Diabetes Research Centre
India	Arun Pande	Lucknow	Lucknow Endocrine Diabetes & Thyroid Clinic
India	Vipul Chavda, Dhruvi Hasnani	Bareja Ahmedabad	Rudraksha Insitute of Medical Sciences
Iran	Hossein Moravej	Shiraz	Avicenna Diabetes Center
Israel	Shlomit Shalitin	Petah Tikva	Schneider Childrens Medical Center of Israel
Kuwait	Doha Alhomaidah	Kuwait City	Farwaniya Hospital
Maldives	Aminath Malha Saeed	Male	Diabetes Association in the Maldives (DAM)
Nepal	Santosh Pokhrel	Butwal	Siddharta Children & Women Hospital (SCWH)
Pakistan	Asher Fawwad	Karachi	Baqai Institute of Diabetology and Endocrinology
South Korea	Jae Hyun Kim	Seongnam	Seoul National University Bundang Hospital
Taiwan	Meng-Che Tsai	Tainan	National Cheng Kung University Hospital
Thailand	Supawadee Likitmaskul	Bangkok	Faculty of Medicine, Siriraj Hospital, Mahidol University
Turkiye	Ilknur Arsanoglu	Duzce	Duzce University Faculty of Medicine
**AUSTRALASIA**			
Australia	Deborah Foskett	Gold Coast	South East Queensland Private Practice Group
New Zealand	Craig Jefferies	Auckland	Starship Paediatric Children's Hospital
New Zealand	Martin de Bock	Ottago	University of Ottago

© 2025 Santova A et al. *JAMA Network Open*.
