# Supplementary material for: Global Inequities in Diabetes Technology and Insulin Access and Glycemic Outcomes
Source: JAMA Netw Open. 2025 Aug 27;8(8):e2528933. doi: 10.1001/jamanetworkopen.2025.28933 (PMC12391998; doi:10.1001/jamanetworkopen.2025.28933)
Supplement: Supplement 2. — Data Sharing Statement [file jamanetwopen-e2528933-s002.pdf]

Data Sharing Statement

Santova. Global Inequities in Diabetes Technology and Insulin Access and Glycemic Outcomes. *JAMA Netw Open*. Published August 27, 2025.
doi:10.1001/jamanetworkopen.2025.28933

Data
Data available: No

Additional Information
Explanation for why data not available: Access to the programming code can be provided by the corresponding author if requested. Due to data protection reasons, data on individual level cannot be provided.
